# Developing an Inpatient Substance Use Disorder Program in a Middle-Income Country: The Experience of Sbrana Psychiatric Hospital, Botswana

**DOI:** 10.1177/29768357251371406

**Published:** 2025-08-31

**Authors:** Anthony A. Olashore, Selebogo M. Moremi, Taboka Maphorisa, Lecha Masego, Mpho L. Hubona

**Affiliations:** 1 Department of Psychiatry, Faculty of Medicine, University of Botswana, Gaborone, Botswana; 2 Department of Clinical Services, Sbrana Psychiatric Hospital, Lobatse, Botswana; 3 Department of Social Work, Sbrana Psychiatric Hospital, Lobatse, Botswana

**Keywords:** substance use, addiction, middle-income country, inpatient program, Botswana

## Abstract

**Background:**

The prevalence of substance use disorders (SUDs) is rising globally, significantly affecting public health. These disorders are associated with reduced quality of life, comorbid mental health conditions, and increased risk of infectious diseases such as HIV. This paper describes the development and implementation of group therapy as an intervention for SUDs in a resource-constrained setting.

**Program Description:**

An 8-week inpatient group therapy program was established at Sbrana Psychiatric Hospital in Botswana. The interdisciplinary team includes a social worker, psychologist, occupational therapist, psychiatric residents, and a psychiatrist. To date, 9 cohorts comprising 55 patients have fully participated. The average age was 35 years; most were males (61%), with alcohol being the most used substance, followed by crack cocaine. Of these, 21 have remained abstinent for at least 3 months and reintegrated into society, 11 have relapsed, and 23 are lost to follow-up.

**Program Insights:**

Challenges encountered include client dropouts, poor reintegration into the community, limited follow-up systems, and financial constraints. Key components contributing to the program’s impact include peer support, relapse prevention, coping strategies, psychoeducation on triggers, and elements of the 12-step recovery model.

**Conclusion:**

Program improvement areas include strengthening follow-up through objective measures, such as urine drug testing, and incorporating structured wellness and recreational activities to support recovery.

## Introduction

Substance use and substance use disorders (SUDs) are increasingly becoming a global health problem affecting a significant portion of the world’s population. This was highlighted by the World Drug Report 2023, indicating that over 296 million individuals used drugs in 2021.^1^ Substance-use disorders are defined in the Diagnostic and Statistical Manual-5 (DSM-5) as a “cluster of cognitive, behavioral, and physiological symptoms indicating that the individual continues using the substance despite significant related problems.”^2^ The related problems include decreased quality of life, suicidal tendencies, and risk of infectious diseases like HIV.^3^ Furthermore, substance use disorders are recognized as chronic, relapsing brain disorders that invade and alter brain reward pathways.^1^ According to the 2021 Global Burden of Diseases, Injuries, and Risk Factors (GBD) 2021 study, substance use disorder contributes significantly to the global burden of disease.^4^ The Disability-adjusted life years (DALYs) attributable to substance use disorders have increased substantially by 74.65% from 1990 to 162,061.67 in the year 2021.^4^ Additionally, it is predicted that the prevalence of these disorders is expected to continue on an upward trajectory in the next 25 years.^4^

## Substance Use Burden in Botswana

The burden of psychoactive substance use, as reported by the United Nations Office on Drugs and Crime, is enormous, with individuals between the ages of 18 and 25 years, who account for 40% of the world population, mostly affected, and Africa accounting for over 60% of these problems.^5^ Botswana is one of the Southern African countries with increasing reports of substance use and related problems, especially among young people.^6-8^ Recent studies highlight that 44.6% of secondary school students in Gaborone reported psychoactive substance use in the past year, with 31.5% meeting DSM-5 criteria for a substance use disorder.^8^ Among university students, lifetime substance use prevalence has been reported at 59.6%, with current use at 37.9%.^7^ A study conducted at Botswana’s national psychiatric referral hospital found that 85.1% of psychiatric inpatients had a history of lifetime substance use, and 63.4% met criteria for a current SUD.^9^ Substance use in Botswana is currently reaching a critical point, with alcohol and cannabis being the most commonly used.^10^ This has been related to an increased burden of psychiatric disorders, including a rise in inpatient admissions.^9^ Whilst it is evident that the burden of addiction is extremely high in Botswana, data concerning the treatment gap remains scarce; however, given the magnitude of the problem and the limited facilities available, it can be hypothesized that the treatment gap is considerably large, potentially much less than the global ratio of 1 in 11; this may reflect the treatment gaps of about 87% observed in low- and middle-income countries.^1,10,11^

## Models of Drug Treatment

Drug treatment models exist in various forms depending on the settings and regions; common types include the medical-disease model, psychological model, and sociocultural model.^12^ The medical-disease models are based on the theory that addiction is a chronic disease with underlying biological causes, such as genetic and neurological changes. This model emphasizes the utilization of medical treatments, which include medication-assisted therapy for alcohol use disorder and opioid use disorder, along with their related behavioral issues.^12^ This model targets detoxification, harm reduction treatment of craving, and relapse prevention; ^12^ albeit these are usually initiated during inpatient treatment, they are often continued as follow-up programs.

The psychological model focuses on the role of coping, emotions, maladaptive behaviors, thoughts, and related behavioral problems in the development of addiction; it employs some evidence-based psychological therapies like brief counseling, psychoeducation, motivational interviewing, interpersonal therapy, 12-step programs, and cognitive-behavioral therapy.^12^ Many of these treatment modalities can be carried out individually and in groups, as well as in outpatient care, depending on the setting, and they address triggers, cravings, as well as behavioral and emotional issues.

The sociocultural model focuses on the role of social and cultural issues in addiction, and these include peer pressure, poverty, family dysfunction, stigma, and discrimination. This model tackles these drug-related social problems with community-driven programs, social support networks, and culturally appropriate interventions, primarily utilizing peer support groups or culturally tailored strategies for specific populations.

The biopsychosocial model integrates medical, psychological, and sociocultural perspectives to establish a comprehensive framework for understanding and addressing substance use disorders. It acknowledges that addiction arises from the interplay of biological factors such as genetic predisposition, psychological components like coping mechanisms and maladaptive behaviors, and sociocultural influences including stigma, socioeconomic challenges, and familial issues. Interventions based on this model are typically multidisciplinary, combining medical treatment, psychological therapies, and community-oriented or culturally appropriate strategies. Research has shown that programs employing this integrated approach tend to yield superior outcomes compared to those relying solely on a single model.^13,14^

## Substance Use Treatment Model in Botswana

Over the years, the most widely practiced intervention in Botswana has been the sociocultural model, primarily implemented by the non-governmental Botswana Substance Abuse Support Network (BOSASNet), which offers community-based intervention programs for outpatient clients. This network provides education, prevention, and rehabilitation services for people suffering from substance use problems. Although this program has assisted many clients over the years, those with severe cases and comorbid psychiatric issues or those who require inpatient treatments like detoxification are referred to South Africa. A few other available private facilities are limited in capacity and primarily serve clients with health insurance. The existing gaps in the treatment program, alongside the necessity for the University of Botswana’s Master of Medicine training, have prompted the establishment of a substance use treatment program within Botswana’s sole psychiatric referral facility. Consequently, this paper aims to highlight the development and implementation of group therapy for individuals with substance use disorders in a resource-constrained environment, with the hope that other settings like ours could learn from our successes and challenges.

## Developing an SUD Program in Botswana

The Botswana National Health Policy outlines the importance of combating alcohol and substance use at multiple levels.^15^ The Ministry of Health committed to making interventions for substance use available in its facilities, but no funding or support has been allocated yet. Nevertheless, the Department of Psychiatry, University of Botswana, decided to rise to this task by floating the idea to start with whatever facility is available. This initiative became imperative due to the rising tide of substance use and its associated mental health sequelae, as well as the necessity to establish a psychiatry residency program in which addiction psychiatry constitutes a vital component of the curriculum. The department started an 8-week inpatient hybrid program, with a psychiatrist, two residents, and a multidisciplinary team that consisted of a social worker trained in addiction, a nurse, and a psychology intern. This initiative provided the first set of our residents with the opportunity to learn about addiction treatment programs. The eligible patients are admitted to the general psychiatric ward and are fetched daily to a place provided by the social work unit of the hospital for their daily activities. The inpatient setting provides an opportunity for intensive treatment, monitoring, and support during a critical recovery phase, as documented in the literature.^16^

## Setting

**Figure 1.**
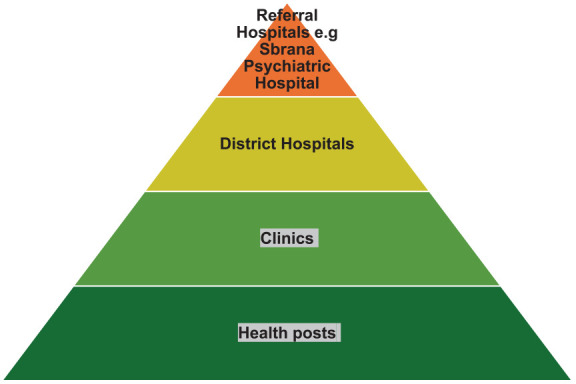
Government health care system.

Botswana is a high-middle-income country in Sub-Saharan Africa with an estimated population of 2.3 million.^17^ Botswana is a landlocked country in southern Africa, bordered by Namibia, Zambia, Zimbabwe, and South Africa. It has a surface area of 581,730 km² (363,581 square miles). As nearly 50% of the population resides in rural areas, this poses a challenge in providing health services to these remote and sparsely populated communities. The government’s health services are divided into a 2-level system: the first is facilities-based, and the other involves community outreach.^18^ The former is organized in a pyramidal structure, with referral hospitals at the top; there are three national referral hospitals, one of which is dedicated to psychiatric care in Lobatse, where the addiction treatment program was established. At the pyramid’s base are health posts and clinics staffed by nursing personnel, who are grouped into clusters under the supervision of a doctor. The pyramidal structure is illustrated in Figure 1. Mental health care services, like others, are mainly funded by the government.

Sbrana Psychiatric Hospital is the sole national referral mental hospital. It is situated in Lobatse, roughly 70 km from Gaborone, the country’s capital. The hospital has a 300-bed capacity.^19^ Clinically, its human resources range from psychiatrists to healthcare auxiliary staff. As of this writing, the facility employs 9 psychiatrists, multiple nurses and doctors, clinical psychologists, social workers, and occupational therapists. It has several wards, including male and female rehabilitation wards, male and female acute wards, forensic wards, an adolescent ward, and a geriatric ward. The rehabilitation wards were originally created to cater to addiction clients, but are presently being used to house stable psychiatric patients since there was no funding to equip them for that purpose. Hence, there is presently no special ward for drug rehabilitation programs.

## Steps in Developing the Program

Prior to the establishment of this program, numerous consultations were conducted: initially, renowned and reputable centers across various countries, including Nigeria, Kenya, and South Africa, were contacted and consulted. Subsequently, all key stakeholders, including hospital management, ward staff, and members of the multidisciplinary team—such as psychologists, occupational therapists, social workers, and dietitians—were engaged. Additionally, local and private facilities, as well as the Ministry of Health through hospital management, were also consulted.

The consultations were subsequently followed by the formulation of a comprehensive proposal, incorporating a needs assessment analysis and report, the Standard Operating Procedure, and essential resources, including personnel, timelines, and various other details. This protocol was developed by the first author (AAO), who possesses substantial expertise and experience in addiction psychiatry, and was submitted to the Department of Psychiatry, the Faculty of Medicine, the management of Sbrana Psychiatric Hospital, and the Ministry of Health.

However, due to delays in receiving feedback, the Department of Psychiatry decided to roll out the program gradually, utilizing existing resources. The program commenced with eclectic group therapy, which served as the exclusive therapeutic intervention for several weeks. It was subsequently supplemented by additional sessions, including individual counseling, psychoeducation, a 12-step program, and other specified components outlined below.

## Description of the Program

The team’s initial plan was to start a 12-week program; however, owing to the unavailability of resources, including a dedicated rehabilitation ward, a limited staffing capacity, and other essential assets, the program has been modified to an 8-week duration. This treatment model employed an eclectic approach, incorporating various aspects such as psychoeducation, 12-step facilitation, occupational rehabilitation, and motivational interviewing. We chose this approach because there’s a broad range of evidence-based treatments available, many of which have proven effective in specialized addiction centers. Research also shows that combining these therapies is more effective.^20^ This approach is well-suited for settings with limited resources, is cost-effective, and can accommodate the diverse needs of group members.^21^

As outlined in Table 1, the program activities include reception and registration, Intake, assessment and investigation, development of a treatment plan, recreational activities, and development of a discharge and follow-up plan. The group meets 3 times a week: on Monday, Tuesday, and Thursday, for 1-hour sessions. Different topics are discussed each week, for example, cues, triggers, and their management, relapse prevention, and occupational rehabilitation. There is a psychoeducation session on Tuesday that covers topics related to issues identified in the Monday sessions, including a basic explanation of how drugs affect the brain, emotional regulation, financial management, and handling interpersonal relationships. On Thursdays, the group discusses the 12 steps of Alcoholics Anonymous, while on other days, they are engaged in various activities, including individual sessions with assigned therapists, reflection, assignments, and recreational activities. During their stay, patients visit the occupational rehabilitation department daily, where they attend the gym and participate in vocational training, such as carpentry and hairdressing. They are also seen weekly in one-on-one sessions with the psychologist based on their needs and issues identified during other sessions by the therapist. In the first 2 weeks of admission into the program, clients’ support systems—including close family members, friends, and colleagues—are evaluated for their family dynamics and integrated into the recovery process. Issues discovered during assessment are addressed and incorporated into their rehabilitation, discharge, reintegration, and the 2-year follow-up plan.

**Table 1.** The Outline of the Rehabilitation Program.

Program phase	Components
Reception and registration	■ Referred clients and relatives are received in the waiting room and are briefly psycho educated on the nature of the program ■ The records office records the client’s details ■ Nursing assessment and ward allocation
Intake	■ Intake screening (If stable clinically, motivated, and ready, proceed to number 2) ■ Clinical history and assessment of clients by the residents within 24 h ■ Investigations, including urine toxicology ■ Administration of tools such as ASSIST^a^ and ASI^b^ ■ Unit consultant review within 72 h of admission.
Treatment Plan	■ Detoxification and treatment of comorbidity^c^ ■ Medical detoxification (1-2 wk) ■ Treatment of psychiatric and medical comorbidities (maintenance of stable mental state) ■ Other regular activities ■ Daily review of the client’s treatment plan by the resident in the unit ■ Weekly review by the multidisciplinary team, including the unit consultant ■ Weekly urine toxicology test for clients^d^
Further assessment and Investigation carried out	■ Personality assessment, by the psychologist ■ Occupational assessment done by an occupational therapist. ■ Home assessment is done by the social worker in the unit. ■ Family meeting
Interventions	■ Psychosocial intervention ■ Group Psychotherapy ■ Individual Psychotherapy ■ Insight-oriented psychotherapy ■ Family therapy, all done by the social workers and clinical psychologists. ■ Occupational intervention: vocational skills acquisition like shoe making, fashion design, barbing, etc
Recreational activities	■ Visit to the gym twice daily (8 am and 4 pm) ■ Game sports daily (football, basketball, table tennis, volleyball, etc.) ■ Indoor games/sports (once daily) are done under the supervision of the health promotion staff of the unit.
At discharge	■ Repeat urine toxicology test on discharge and monthly

a Alcohol, Smoking, and Substance Involvement Screening Test.

b Addiction Severity Index.

c Done in their respective wards.

d Not done regularly due to a lack of necessary tools.

## Success

**Figure 2.**
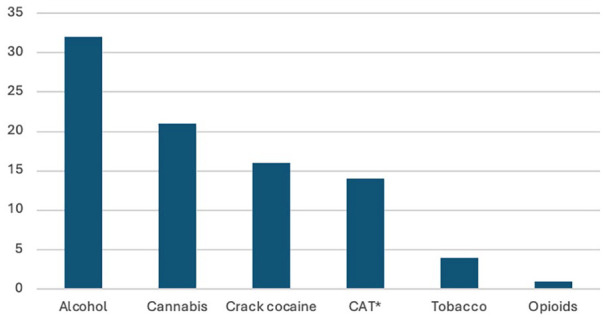
Pattern of psychoactive substance use in patients who completed the program. *Methcathinone.

Since the program’s start in March 2023, a total of 61 patients have been enrolled across 9 cohorts; of these, 55 patients have received treatment, while only six clients have dropped out within 2 weeks of admission. Consistent with global trends,^22^ of the 55 who completed the program, the majority were male, comprising up to 61% of the group. The average age was 35 years, with the oldest patient being 55 years old and the youngest being 21 years old. Most patients (60%) were unemployed. The most commonly used substance was alcohol (32, 58.1%), followed by cannabis (21, 38%), and crack cocaine (16, 29%) (Figure 2). Twenty-three patients have been lost to follow-up, 11 have relapsed, and 21 are abstinent (not using drugs for at least 3 months) and have reintegrated well into society; some have successfully returned to work/got employed, while others have started small businesses (Table 2). Most patients who relapsed and were lost to follow-up were males (63%), the average age was 33 years, and the common substances used by this group were crack cocaine (63%), alcohol (30%), and multiple substances (60%). We do not have accurate information on the status of those lost to follow-up, even though some of them may be abstinent; only those who are committed to regular clinic appointments and have returned to work or are employed are considered to be doing well.

**Table 2.** Patient Demographics and Outcomes Overview.

Characteristics	Number of clients	Predominant sex	Average age (years)
Total intake	61	Males (61%)	35
Dropped out within 2 wk	6	Males (50%)	27
Completed program	55	Males (61%)	35
Relapsed	11	Males (63%)	30
Lost to follow up	23	Males (60%)	33
Doing well^a^	21	Females (59%)	35

a 13 went back to work, seven got employed, and one went back to school.

## Challenges

### Financial Constraints

The program was started without any funding. Therefore, some necessities could not be obtained. The program had no allocated ward, and clients were recruited from the existing rehabilitation wards, which housed patients with a wide variety of disorders. The mixing of patients meant that during their stay in the hospital, they were exposed to other patients who were not in the program and still using substances, and in some encounters, smoking in the wards.

Due to a lack of funds, we are unable to acquire recreational activity items, and patients have limited options for leisure activities. We are also unable to obtain objective measuring tools, such as urine drug tests.

### Re-Integration into Society

After their hospital stay, the patients are discharged back into society. Due to the staff shortage, there is no follow-up on how they are coping at home and in their workplaces.

### Lack of Follow-Up

After discharge, some patients fail to attend the monthly outpatient sessions and are subsequently lost to follow-up. There is a lack of substance use counselors in some regions of the country; therefore, those who are unable to attend the monthly reviews are not reviewed.

### Client Dropouts

Some clients left the program because they were not satisfied with the living conditions in the hospital. Some clients displayed behavioral issues and were expelled from the program. Other participants were removed from the program due to their inability to follow up on the discussions, particularly those with a diagnosis of intellectual disability.

### Homelessness

Some clients were homeless, complicating social support and recovery.

## Discussion

This paper reflects on and describes the process of developing an addiction program in a setting with low resources and limited support from significant stakeholders. The substance use treatment program uses the existing facilities and resources, such as the ward and the staff who are interested in addiction treatment, including those who are not specifically trained for that purpose. The program is based on the integration of 3 established models: biological, psychological, and social, to deliver holistic addiction care.

In Botswana, a developing country with a nascent mental health system, resources allocated to mental health care are typically limited.^19^ As previously mentioned, only a few outpatient, publicly owned addiction treatment centers existed in Botswana for several years, with most patients requiring comprehensive treatment being sent to South Africa. After years of several unsuccessful efforts made to establish a government-owned comprehensive facility, the program kicked off over 2 years ago, based on Arthur Ashe’s quote: “Start where you are, use what you have, do what you can.”^23^ The University of Botswana’s psychiatry department initiated group therapy in response to the increasing prevalence of substance use disorders, which were compounded by insufficient resources. Despite these constraints, utilizing the available resources was deemed essential for addressing the escalating issue of substance misuse.

### The Intervention Model of the Substance Use Treatment Program

The program is based on the biopsychosocial model, which has been associated with improved abstinence and retention outcomes compared to single-model approaches.^24^ While the biological model is used to some extent, its implementation is limited by the lack of specialized infrastructure. Detoxification is conducted in the general ward, using medications such as benzodiazepines and thiamine for the treatment of alcohol withdrawal. Additionally, naloxone is utilized for the management of opioid intoxication, whereas nonsteroidal analgesics, clonidine, metoclopramide, loperamide, and diazepam are used for the treatment of mild opioid withdrawal, under the supervision of the psychiatric team. Unfortunately, other medications used globally to treat addictive disorders, such as methadone for opioid withdrawal, disulfiram as an alcohol deterrent, acamprosate for alcohol dependence, and nicotine patches for nicotine withdrawal, were not used due to their unavailability within our system setting.^25^ Despite these constraints, the program has achieved a 90% success rate in biological detoxification. Nonetheless, in our program, pharmacological management is usually, but not always, followed by psychological intervention, as practiced elsewhere.^26,27^

As previous authors suggest, the psychological model, which focuses on non-pharmacological or biological interventions, is the core of our program.^12^ This begins with the assessment of the clients’ readiness for treatment using the Wheel of Change^28^ and motivational interviewing. As has been reported, this helps resolve ambivalence in clients observed to be at the contemplation stage, moving some clients from this point to the point of action.^29^ Although the rate of success has not been objectively measured, the unreported response rate among the few clients who initially expressed ambivalence or reluctance has been commendable. The role of psychoeducation has been widely reported as one of the main tools in the treatment of individuals with addiction problems,^30,31^ and this has been the core of our program. This addresses the key areas shown to be pertinent in addiction treatment as reported by Magill et al;^30^ these include:

■ Enhancing understanding of addiction: Clients are educated on the neurobiological basis of addiction to reinforce that it is a disease, not simply a matter of willpower.
■ Identifying root causes and building coping skills: Many clients report using substances as maladaptive coping mechanisms. Through psychoeducation, they learn relapse prevention strategies and build self-efficacy.
■ Understanding the value of social support: Clients are encouraged to recognize and make use of available support networks to reduce isolation and enhance recovery.
■ Increasing awareness of the consequences of continued use: Education about the risks of drug use serves as motivation for change.
■ Addressing stigma: The program helps clients understand the role of public, self, and structural stigma and encourages a reframing of addiction as an illness rather than a moral failing.

In addition to psychoeducation, individual counseling is provided to address personalized issues identified by the client or therapist. An occupational therapist offers assessments and counseling, typically once a week or as needed by the clients. As reported by many centers, occupational stability may be compromised due to the effects of substance use, which can lead to absenteeism, reduced productivity, accidents, and injuries, which may ultimately result in dismissal.^32^ It has also been widely reported that clients with substance use disorders are unemployed or have difficulty securing gainful employment.^32^ Our occupational therapist helps identify strengths, values, interests, and challenges while developing recovery plans that emphasize the importance of engaging in meaningful daily activities. Of the 21 clients who reported work-related problems or identified themselves as unemployed upon admission, 13 returned to work, and 8 were able to find gainful employment or resume their education after completing 8 weeks of occupational therapy (Table 2).

One essential type of psychotherapeutic intervention frequently preferred within our program is the eclectic approach, as it is suitable for optimizing outcomes while potentially reducing costs through strategic resource allocation and customized solutions.^26,27^ It is flexible and easy to use with resources available in our setting, and importantly, it can be tailored to individual needs.^27,33,34^ Additionally, this approach may incorporate various therapeutic modalities; consequently, it can address multiple facets of addiction, including underlying psychological issues that may not have been previously reported.^27,33,34^ One important example is art therapy, which clients use to express thoughts and feelings that may be hard to put into words; ^35^ our program benefits from this activity, as an intern with repeated contract renewals facilitates it once a week in a group setting for our clients during their 8-week programs.

Furthermore, the 12-step program constitutes a fundamental aspect of the psychological model employed by our program, which is administered weekly in a group format. According to Humphreys et al,^36^ it underscores the significance of acknowledging addiction as a disease that can be managed yet never wholly eradicated. This approach fosters individual maturity and spiritual development, reduces self-centeredness, and facilitates assistance to others who are grappling with addiction.

Complementing these psychological approaches is the social model, which highlights the role of social factors in both the development and treatment of substance use disorders.^12^ As reported in the literature,^37^ some of the social factors that were reported by our clients include social stressors relating to family, relationships, and work, peer influence, poverty, discrimination, and modeling. These are among the social issues addressed during this program, both in the inpatient and outpatient sessions. Fortunately, we have a social worker who is trained in addiction treatment. During the patient’s admission, the social worker performs an occupational assessment and a home assessment, which involves evaluating the existing social network. The social worker identifies detrimental family dynamics (eg, High Expressed Emotions), a stressful work environment, and the presence of stigma, particularly within the community context. The social worker collaborates with both the family and the client to formulate treatment and aftercare plans; to facilitate this process, families are encouraged to participate in sessions with the clients throughout and at the conclusion of the program, as this has been shown to enhance retention in care and support sustained sobriety.^37^ The aftercare follow-up is conducted by social workers, who call the client and their relatives by phone to assess and monitor progress. They also participate in the monthly outpatient programs, which include all the members of the multidisciplinary team and, occasionally, a significant family member.

### The Treatment Setting of the Substance Use Treatment Program

While the program adopts individualized modes of treatment, it places special emphasis on group intervention and a peer-centered approach. Over the past two years, the team has treated 55 clients in groups of 6 to 10 members, which is within the commonly reported size for group therapy;^38^ however, this is also related to the limited facility and our dropout rate. These groups were composed of individuals dependent on various substances, including alcohol, marijuana, methcathinone, cocaine, and opioids. According to Yalom’s therapeutic factors,^39^ cohesiveness is crucial for effective group therapy. We observed the development of this cohesiveness within the groups over time, as participants shared a strong sense of unity. The emphasis was placed on the notion that addiction is not solely about the substance itself ^40^ but about the behavioral patterns and emotional struggles common to all participants. However, the inclusion of individuals with diverse substance dependencies presents certain challenges, such as differences in recovery trajectories, varying pharmacological needs, and the potential for substance-specific stigma. For example, individuals with alcohol dependence may perceive themselves as more “successful” than those struggling with cocaine or opioid addiction. Nevertheless, in our experience, this has not been a significant issue, and the mixed-group model continues to be effective due to the participants’ readiness for recovery, regardless of the substance to which they were dependent; furthermore, the small group size facilitates more manageable interactions.

### The Duration of the Substance Use Treatment Program

Ideally, the duration of inpatient addiction treatment should extend for a minimum of 3 to 6 months, accompanied by a follow-up period of no less than 12 months.^41^ However, due to inadequate financial support and a lack of infrastructure and human resources within our context, we have implemented an 8-week program, supplemented by an intensive outpatient follow-up that extends beyond 12 months. Our psychoeducational sessions were limited to once weekly, with additional psychological interventions provided on an individual basis. Additionally, the 12-step program was limited to once per week, whereas interactive sessions for occupational therapy occurred twice per week and were complemented by daily supervised practical occupational therapy sessions. The frequency of individual sessions varied depending on the availability of attending psychologists or social workers, which may have implications for addressing the core issues related to the onset of substance use. Even though studies have shown that longer duration of treatment confers greater benefits and outcomes,^41^ we have recorded a noticeable success in our program. Perhaps this could be explained by the severity of our enrollees, since those with severe substance use disorders have been documented to require a longer period of treatment.^41^ Our family intervention programs and intensive follow-up program, which involve regular phone calls and an extended follow-up period, may also have contributed to the program’s success thus far.

### Discharge and Follow-Up in the Substance Use Treatment Program

Our program incorporated a structured yet flexible follow-up plan, which was initiated early during the patient’s hospital stay to prepare for discharge. This process included family counseling sessions designed to repair relationships and facilitate the patient’s reintegration into the community. Each patient was also provided with an individualized relapse prevention plan, identifying personal triggers and coping strategies, which is a key element in maintaining sobriety. Although monthly follow-up appointments were scheduled for at least 2 years post-discharge, financial constraints have posed a challenge for some participants. Additionally, we do not currently offer maintenance medications for certain types of substance dependence, which could be beneficial in some cases. Urine drug screenings, which are recommended in the literature for monitoring abstinence during follow-ups,^42,43^ have not yet been implemented but are planned for future integration into the program. Our assessment to date has been predominantly subjective, encompassing reports from family and friends, successful employment, enhanced mental well-being, positive physical health, and a commitment to monthly follow-ups, as previously reported in the literature.^44,45^

It is noteworthy that our observations suggest women tend to achieve greater long-term success compared to men, as previously reported;^46^ this may be attributed to their propensity to establish supportive and close relationships with friends and others. Such relationships are associated with enhanced psychological well-being and facilitate the coping process during adverse events, including substance use disorder. Additionally, the health-seeking behaviors frequently observed in women, as opposed to maladaptive coping strategies more common among men, may have also contributed to the more favorable outcomes observed in women.^46^ We also noted that young people tend to leave the program early, possibly due to low motivation, drug cravings, the absence of alternative recreational activities, or dissatisfaction with the program. Furthermore, adolescence and young adulthood are times of major change, which can complicate managing the intricacies of addiction treatment.^47^

## Conclusion

Despite the challenges faced, the program has achieved several successes. We have successfully assisted approximately 55 of the 61 patients enrolled in the program. Although it remains resource-constrained, the program is supported by a multidisciplinary team comprising psychologists, doctors, psychiatrists, occupational therapists, and social workers, all of whom collaborate to deliver the best possible care to the clients. Key elements of the program, including psychoeducation on triggers, coping mechanisms, relapse prevention, peer support, and 12-step integration, are in place. Moreover, the underlying comorbidities of participants are also diagnosed and managed. However, there is still room for improvement in providing more structured recreational and wellness activities, as well as enhancing follow-up with objective monitoring methods, such as urine drug screenings.

## Insights and Recommendations

### Improving Retention

○ Offering support for clients with co-morbidities could improve retention. This could involve collaboration with other healthcare professionals to address mental health or physical health conditions that interfere with recovery.
○ Additional counseling or peer support groups could help address behavioral challenges and encourage clients to stay engaged with the program.

### Social Support Systems

○ Partnering with housing services or social welfare organizations could provide more comprehensive support for homeless clients.
○ When feasible, involving families or significant others in the recovery process could strengthen clients’ support networks.

### Addressing Financial and Logistical Barriers

○ Offering transportation or financial assistance to clients who live far from the hospital could improve attendance.
○ Telehealth follow-ups or remote counseling might also be an option to consider, especially for clients in remote areas.

### Expanding Follow-up Care

○ The lack of substance use counselors in some areas is a significant barrier. Training more counselors or collaborating with existing local services could create a wider network of support for patients’ post-treatment.
○ Creating a system of mobile counselors or community-based peer recovery coaches might also bridge the gap for those in underserved regions.
